# Characterization of Three Types of Recycled Aggregates from Different Construction and Demolition Waste: An Experimental Study for Waste Management

**DOI:** 10.3390/ijerph20043709

**Published:** 2023-02-19

**Authors:** Pablo Saiz Martínez, Daniel Ferrández, Alberto Melane-Lavado, Alicia Zaragoza-Benzal

**Affiliations:** 1 Department of Financial Economics, Accounting and Modern Language, Rey Juan Carlos University, Paseo de los Artilleros, s/n, 28032 Madrid, Spain; 2 Departamento de Tecnología de la Edificación, Universidad Politécnica de Madrid, Avenida Juan de Herrera 6, 28040 Madrid, Spain

**Keywords:** physicochemical characterization, recycled aggregates, construction and demolition waste (CDW), homogeneity

## Abstract

Achieving sustainable management and efficient use of natural resources stands out as one of the goals included in the Goals for Sustainable Development in the 2030 Agenda. The construction sector is currently far from presenting an efficient model in terms of treating waste generated by it. Variations in the physical and chemical properties of recycled aggregates coming from construction and demolition waste are one of the main reasons of their limited use in the production of construction materials. This research presents a physicochemical characterization of three different types of recycled aggregates coming from different types of waste: concrete, ceramic and mixed. Physical characterization shows that recycled concrete aggregate has better physical properties compared with mixed recycled aggregate and ceramic recycled aggregate, which makes it more suitable for use in masonry mortars and concrete, due to its higher dry density (2210.33 kg/m^3^), its lower content of fines (5.17%), its lower friability coefficient (24.60%), and its water absorption coefficient (6.70%). Chemical characterization shows that none of the tested recycled aggregates contains traces of harmful chemical agents that exceed the limits established by the reference regulations. Finally, the statistical analysis shows good homogeneity for these raw materials, obtaining low coefficients of variation and values within the recommended in each of the calculated confidence intervals.

## 1. Introduction

Nowadays, one of the main challenges is to make possible a rational use of resources by the current civilization satisfying its development, without impacting the ability of future generations to satisfy their own needs [1]. For this reason, the United Nations Organization has included 17 Sustainable Development Goals within the 2030 Agenda that allows progress towards a more prosperous development worldwide [2]. A good part of these goals will be achieved if sustainable waste management and efficient use of natural resources is achieved [3]. The efficient management of waste becomes complicated in some industrial sectors such as construction, where the generated waste is of very heterogeneous nature and it is difficult to separate it at source [4]. Because of these deficiencies, there are adverse environmental effects such as contamination of soils and aquifers, landscape deterioration, or excessive elimination of resources without the possibility of reuse [5].

Thus, the construction sector continues to be the area of industrial activity with the fewest number of environmental management systems [6]. Specifically in Spain, the rate of recovery of Construction and Demolition Waste (CDW) stands at 75%, which is below the European average of around 90% [7]. However, the proper management of CDW is a key challenge in the global defense to achieve a model based on the circular economy [8]. In this way, one of the main applications of the CDW is the manufacture of recycled aggregates that serve as raw materials for the fabrication of construction materials and their use in construction systems [9]. The aggregates are the most widely used raw material in the construction sector [10], being these granular materials the second most consumed natural resource in the industry after water [11]. In addition, the health crisis caused by the SARS-CoV-2 virus has caused the closure of multiple power plants and aggregate extraction mines, which increases the need to invest efforts in improving the management of the CDW for its reincorporation into the production process [12]. 

However, despite the relevance of recycled aggregates as raw material and the regulatory efforts that reward their use [13], at present, their applications in the building are quite limited and their use is mainly focused on the elaboration of prefabricated buildings and roads [14,15]. One of the main challenges faced by aggregate recycling plants is the high heterogeneity of the CDW used in their manufacture [16], which is collected in construction and demolition works and transported to the waste stations to be treated for subsequent separation, crushing, grinding, and removal of impurities by visual inspection [17]. 

Thus, the origin of waste that reaches the recycling plant, the nature of the screening processes and the procedures to eliminate its impurities, are the main causes for differences between the physical and chemical properties of recycled aggregates coming from construction [18]. Regarding the quantity of adhered mortar present in this type of aggregate, values around 49.50% have been obtained for aggregate sizes between 4/16 mm [19], although it is true that this quantity of mortar decreases after crushing [20]. The grinding process in turn has a direct influence on the granulometry of the aggregates, which affects properties such as workability, porosity, permeability, or the degree of compaction [21,22]. Some authors have studied this granulometry to determine the percentage of fines generated during the handling of the coarse aggregate, which varies from 0.27% to 1.14%, and has a direct impact on the amount of mixing water necessary to work with these materials [23].

The crushing process impacts as well the shape and the surface texture of the aggregates. It can be observed that the aggregates with more rounded shape present better properties in terms of workability and mechanical resistance, when the aggregates with angular shape and uneven surface present better adherence properties [24]. The Spanish regulations establish a maximum limit of 35% for the index of slabs in aggregates for concrete, while there is no limitation for the manufacture of masonry mortars [25]. On the other hand, recycled aggregates generally have a lower density and higher absorption than natural aggregates [26,27], and there are also discrepancies regarding these values since they depend on the origin and type of crushing that the aggregate has suffered [28,29]. Resistance to fragmentation is an indicator of the resistance to wear and abrasion of aggregates, which in the case of recycled aggregates presents lower values than those obtained in natural aggregates, due to the weight loss that occurs after eliminating a part or all the adhered mortar [30]. There are also differences regarding the impurity content of these aggregates, as depending on their origin and use, some values or others will be admissible [31]. The most studied chemical compounds for their direct influence on mortars and concretes are chlorides and sulfates [32].

Therefore, several physical and chemical properties can condition the use of recycled aggregates in the construction sector. Unlike other research, in this paper, three different types of aggregates have been used to determine their properties, which can be classified according to the origin of the waste that generates them: recycled concrete aggregates, recycled ceramic aggregates, and mixed recycled aggregates. Recycled concrete aggregate is obtained from recycling concrete waste with a low content of ceramic and bituminous particles (it is considered recycled concrete aggregate if it contains more than 90% of this material) [33]. Recycled ceramic aggregate with ceramic material content exceeding 90% is characterized by its higher absorption and lower density [34]. Finally, mixed recycled aggregate comes from the treatment of different types of waste and has a content of ceramic and concrete particles of less than 20% and 90%, respectively [35]. All the aggregates used in this research come from two fixed recycling plants that process around 600 t/h of waste from different works in the city of Madrid. Moreover, they have a permanent installation of separation belts, surplus material removal equipment, and shredders for the different types of aggregates [36].

The objective of this research is to study the homogeneity in terms of physical and chemical properties of recycled aggregates, and to verify that these properties comply with the requirements of current regulations for their use as construction aggregates. This paper is innovative, as currently there is no exhaustive research work in Spain that compares properties of recycled concrete aggregates, mixed recycled aggregates and recycled ceramic aggregates. For this, an experimental campaign has been conducted during two years, taking monthly samples from independent production processes, and the most relevant properties that these materials must meet for their use as raw material in the sector of construction have been analyzed. This research intends to synthesize in a rigorous and accessible way in a single paper the experience obtained during the characterization of the three different types of recycled sand most used in construction.

## 2. Methodology

This work has been conducted for two years in collaboration with the “La Palentina” and “Molar” integral waste treatment plants, both located in the Community of Madrid, Spain. These recycling plants have made it possible to monitor the characterization of the waste by providing monthly samples of the three types of recycled aggregates analyzed in this research by obtaining a total of 24 samples of each aggregate. These samples were collected periodically every month for a total time of two years. The aggregates used for the physicochemical characterization have been recycled concrete aggregate (RA–Con), recycled ceramic aggregate (RA–Cer), and mixed recycled aggregate (RA–Mix), complying with the classification that is included in the Spanish guide to recycled aggregates (GEAR) [34]. Furthermore, all the used samples have been chosen following the criteria of having been generated from independent aggregate production processes, in order to study the possible variability in the main properties of this material. 

**Figure 1:**
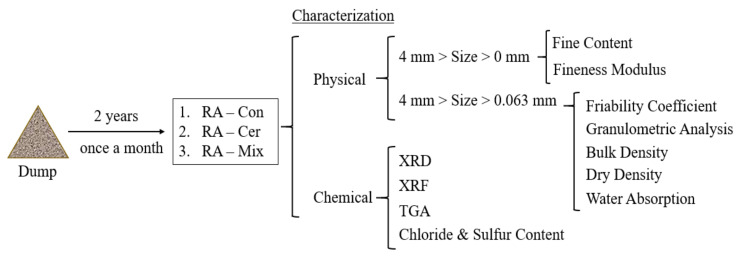
Schematic summary of the tests conducted in this research.

The experimental program conducted in this research can be divided according to the nature of the tests on recycled aggregates: one part for physical characterization and the other for chemical characterization. On the other hand, the Statgraphics Centurion software was used for the processing and statistical analysis of the results. Schematically, this classification can be observed in Figure 1.

### 2.1. Physical Characterization

**Figure 2:**
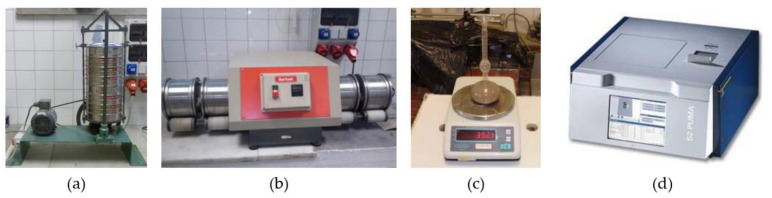
Some of the equipment used for the work: (**a**) Back-and-forth sieve shaker for fines content and fineness modulus; (**b**) Equipment for determining the friability coefficient; (**c**) Bulk density test; (**d**) X-ray fluorescence equipment.

The different physical characterization tests have been conducted on recycled aggregate samples of sizes included between those retained in the 0.063 mm mesh light sieve and those that pass through the 4 mm mesh light sieve, except for the properties of analysis of the fine content and the fineness modulus in which all the granulometry that pass through the 4 mm sieve have been included (Figure 2). In all the mentioned cases, a reciprocating mechanical sieve has been used. It is important to note that various investigations have shown the poorer quality of fines in recycled aggregates, which negatively impacts the preparation of mortars and concretes [37,38].

All the tests conducted in this research have been performed at the Polytechnic University of Madrid and the Spanish regulations applicable to each one of them have been followed, as they are current and applicable at state level. These regulations have been drawn up in the Technical Standardisation Committees and, wherever possible, the official Spanish version of the European UNE standards has been used. It should be noted that there are other standards (such as the ASTM standards), which should be taken into consideration when conducting a similar case study in other geographical regions in order to adapt the tests presented in this work.

For the three types of recycled aggregates used in this research: RA–Con, RA–Cer, and RA–Mix, the following tests have been conducted: fines content (UNE-EN 933-1), fineness modulus (UNE-EN 13139), friability coefficient (UNE-EN 146404), granulometric analysis (UNE-EN 933-1), bulk density (UNE-EN 1097-3), dry density (UNE-EN 1097-6) and water absorption (UNE-EN 1097-6). The tests performed on the aggregates have been chosen based on the impact of these characteristics on the most relevant properties of mortars and concrete [39].

For the statistical analysis of the results obtained for the different physical properties tested, it has been verified that all the physical variables analyzed behave according to a normal distribution. To do this, the Shapiro–Wilk test for small samples (<50) was performed and its results are summarized in Table 1. In the first stay, all the tests performed allowed to verify the normality of the variables except for the corresponding tests to ceramic aggregate and mixed aggregate in water absorption property. In both cases, the sample size was expanded to 36 samples, thus complying with the normality hypothesis. It should be noted that the Shapiro–Wilk test shown in Table 1 is based on the comparison of the quartiles of the normal distribution adjusted to the data for each of the various tests conducted on three types of tested aggregates. When the *p*-value is higher than or equal to 0.05 (significance level), it means a normal distribution with a level of 95% confidence [40].

Once the normality of the variables has been verified, confidence intervals have been built to analyze the maximum and the minimum values that different tested recycled aggregates can reach in terms of different properties with a confidence of 99.7%. 

### 2.2. Chemical Characterization

Regarding the chemical characterization of the studied construction and demolition waste, the following tests have been conducted: X-ray diffraction using a Siemens Krystalloflex D5000 equipment (Berlin, Germany), with a graphite monochromator with Cu-Kα and X-ray fluorescence with the help of a spectrometer Bruker S2 Puma (Karlsruhe, Germany) for each of the 72 collected samples, and analysis of the chloride content and total sulfur content following the recommendations of the standard UNE-EN 1744-1:2010+A1:2013, where six samples of each type of aggregate were analyzed. To complete this chemical analysis, a thermogravimetric analysis has also been performed on each of the recycled aggregate typology included in this research with the help of a thermogravimetric analysis equipment SDT Q600 from TA Instruments (New Castle, DE, USA).

This chemical analysis allows obtaining information about the composition of each of the three types of aggregates in order to better understand their properties and observe their variability, although it is true that current regulations are less restrictive in terms of chemical composition than in terms of physical characterization. The only exception would be the chloride and sulfur content, which are limited in order to use this type of recycled aggregates in the manufacture of mortars and concrete [41].

## 3. Analysis of Results

### 3.1. Study of the Physical Properties of Recycled Aggregates

#### 3.1.1. Fine Content

**Figure 3:**
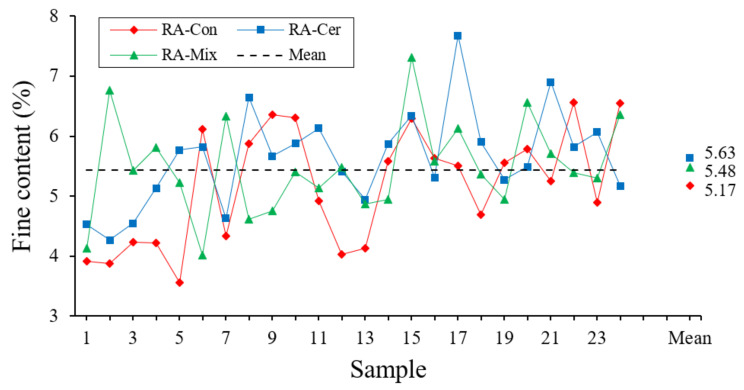
Fine content expressed as a percentage for each of the aggregate samples analyzed.

The percentage of fine particles has been determined following the recommendations of the UNE-EN 933-1 [42] standard. Sieving is conducted for each sample of desiccated aggregate using a series of sieves with mesh lights ordered from highest to lowest of 4, 2, 1, 0.5, 0.250, 0.125, 0.063 mm, respectively. Thus, the content of fines is expressed as the percentage of aggregate that passes the 0.063 mm sieve and is retained at the bottom, concerning the total mass of aggregate sieved in each sample. The results obtained for the 72 samples can be observed in Figure 3.

As it can be observed in Figure 3, the different tested samples have obtained fines content values close to the average value for the three types of recycled aggregates. These values correspond to those obtained in other investigations [43,44], where it was highlighted that the existence of fines in aggregates directly affected the properties of mortars and concretes made with them, such as the decrease in the mechanical resistance, the lower workability of the dough in a fresh state and the lower durability of the used material. 

Table 2 shows the results derived from the statistical analysis.

Table 2 shows the existence of a single homogeneous group for three types of aggregates, which demonstrates that the content of fines is not significantly different for any of the used recycled aggregates.

On the other hand, the coefficient of variation obtained is similar in all tested recycled aggregates and its value does not imply high heterogeneity, mainly due to the similarity in the manufacturing processes for the aggregates from both recycling plants. Finally, according to the UNE-EN 13,139 [45] standard, which regulates the maximum fines content for the mortar preparation, the analyzed in this research on recycled aggregates would be classified as Category 3 in all the calculated confidence intervals; that is suitable for masonry mortar fabrication.

#### 3.1.2. Fineness Modulus

**Figure 4:**
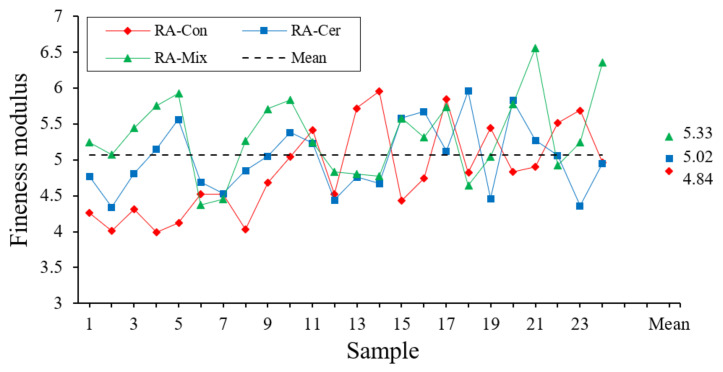
Modulus of fineness obtained for each aggregate type.

The fineness modulus is intended to classify aggregates based on their granulometry. This property directly affects the amount of mixing water necessary to achieve a proper workability and therefore the final resistance that mortars and concretes can achieve [46]. To determine the fineness modulus of the different recycled aggregate samples, the procedure described in the UNE-EN 13,139 standard [45] has been followed. In this way, the fineness modulus is calculated by adding the retained and accumulated percentages of the sieves with mesh sizes 4, 2, 1, 0.5, 0.250, 0.125, 0.063 mm dividing by 100. The results corresponding to the fineness modulus can be observed in Figure 4.

In the case of the fineness modulus, Figure 4 shows even more grouping of the data around the average value, exceeding the recommended values for three types of studied aggregates (2.5–3). On the other hand, the statistical analysis shown in Table 3 has also been conducted, where the mean value and the standard deviation obtained in this property are presented for each of the studied recycled aggregates, observing a low and very similar coefficient of variation in the three tested materials. In the multiple range test, a significant difference is observed between RA–Con and RA–Cer, and it can be concluded that recycled concrete aggregate has a lower fineness modulus.

#### 3.1.3. Friability Coefficient

**Figure 5:**
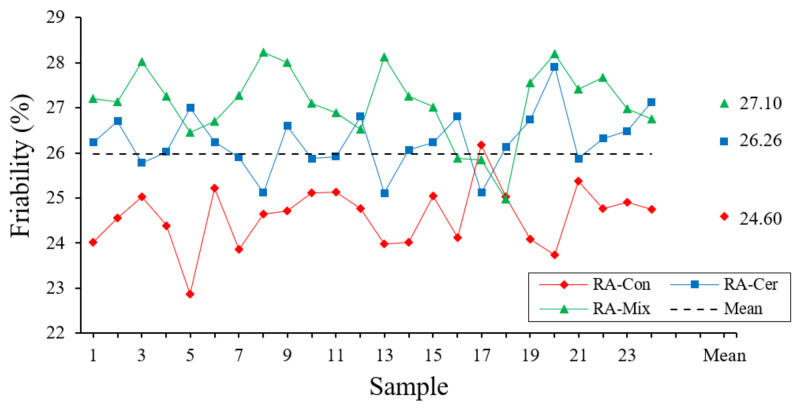
Friability coefficient for aggregates expressed in percentages.

The determination of the friability coefficient of the aggregates has been conducted following the recommendations of the UNE-EN 146,404 [47] standard. The friability of aggregates is defined as the resistance that an aggregate present to its degradation and breakage of its vertices and edges by the impact. To conduct this test, a 200 mm diameter rotation cylinder (Micro-Deval apparatus) is used under standard abrasion conditions. The cylinder is started in conditions of rotation of 100 turns at 1500 rpm with a mass of aggregate of 500 g previously dried, which is introduced into the cylinder together with a load of steel spheres of mass 2.5 kg and 2.5 L of water. Once the rotation is completed, the sample is sieved to remove fines and the coefficient of friability is calculated as the relation between the initial weight of the sample and the weight of fines obtained after the test. The results of friability coefficient of the aggregates are shown in Figure 5.

As observed in Figure 5, values of aggregates friability of around 26% have been obtained. This value has an impact on the final properties of mortars and concretes elaborated with these recycled aggregates, since, although it can improve the workability of the mix in a fresh state, it impacts negatively the compressive strength of the hardened sample and causes the decrease in its elasticity modulus [48]. These effects have been verified in other previous investigations, where it has been observed that mortars fabricated with aggregates that had a higher coefficient of friability impacts negatively compressive strength and elasticity [49].

The results derived from the statistical analysis are shown in Table 4.

The results of the statistical analysis collected in Table 4 show a significantly lower coefficient of friability in case of recycled concrete aggregates red with other two types of recycled aggregates included in this research. This characteristic has a positive impact on mechanical behavior of mortars and concretes fabricated with this type of aggregate, as shown in various investigations [19]. The variability presented in three types of aggregates is low and although with values higher than those commonly obtained for natural aggregates, the obtained values are lower or equal to 40% which is recommended by the UNE 83115: 1989 EX standard [50].

#### 3.1.4. Set Density

**Figure 6:**
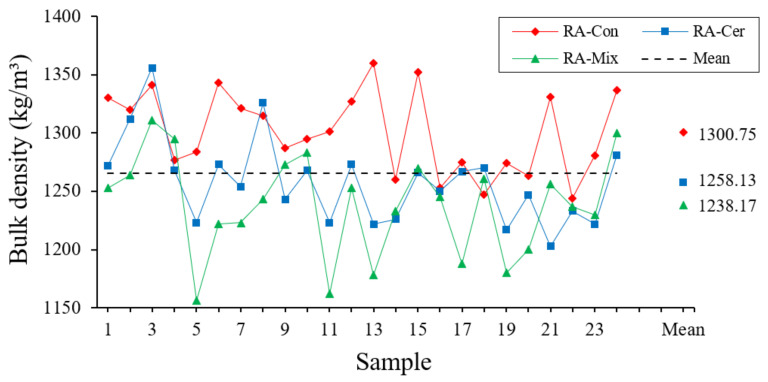
Results obtained for Bulk Density (kg/m^3^).

To determine the overall density of the recycled aggregates included in this research, the recommendations of the UNE-EN 1097-3 standard [51] have been followed. The overall density is obtained as the quotient between the weights of the dried aggregate that fills a container of a determined volume, by the volume of the container. The results of this test are shown in Figure 6.

The results derived from the statistical analysis for this property are shown in Table 5.

Statistical analysis observed in Table 5 shows that there are significant differences in the formation of homogeneous groups in case of recycled concrete aggregates compared to recycled ceramic and mixed aggregates that present a lower density on average. These results are consistent with other previously conducted research where the beneficial effect of this lower density of recycled ceramic aggregates as impact noise attenuators could be verified [52]. In addition, analyzing the coefficient of variation and the normal probabilistic graph, homogeneity in the data for three types of tested aggregates can be seen.

#### 3.1.5. Relative Density

**Figure 7:**
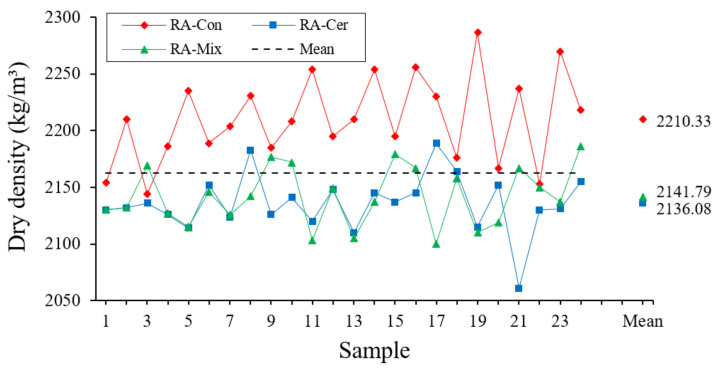
Results obtained for Dry Density (kg/m^3^).

To determine the relative density of the recycled aggregates, the recommendations of the UNE-EN 1097-6 standard [53] have been followed. Relative density has been obtained by dividing the mass of the aggregate previously dried by the volume of water displaced by the aggregate when introduced into a pycnometer. The results corresponding to the relative density of three types of analyzed aggregates are shown in Figure 7.

In Table 6, the results derived from the statistical analysis for this property are shown.

Relative density values observed in Table 6 show that there are low coefficients of variation in three types of tested recycled aggregates. The relative density values in case of aggregates from concrete waste are significantly higher than those observed in the samples fabricated with recycled ceramic and mixed aggregates, as can be seen in the multiple range tests.

On the other hand, it is necessary to highlight that this higher relative and overall density corresponding to recycled concrete aggregates, has a positive impact on mechanical behavior of mortars and concretes fabricated with this aggregate as a raw material [54]. Some research works have corroborated how the higher density of recycled concrete aggregates influences directly the increase in the compressive strength of materials fabricated with these aggregates and causes the increase in their modulus of elasticity [55]. Another property that is related to density is the durability of the material. The durability is higher when the density of finally prepared mortar or concrete is higher [56].

#### 3.1.6. Water Absorption

**Figure 8:**
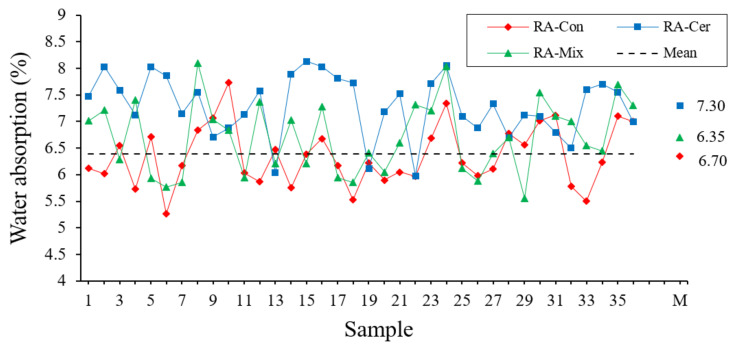
Water Absorption Coefficient obtained for the aggregates expressed in percentage.

To determine the absorption of recycled aggregates, the recommendations of the UNE-EN 1097-6 [53] standard have been followed. Absorption coefficient is calculated according to the following expression (1):

(1) $$WA=\frac{\left(M_{S}-M_{D}\right)}{M_{D}}\cdot 100$$

where *M_S_* is the mass of the saturated and surface-dried aggregate, weighed in the air in grams, and *M_D_* is the mass of the test portion dried in an oven and weighed in the air in grams. The results of this test are shown in Figure 8.

In Table 7, the results derived from the statistical analysis for this property are shown.

Table 7 shows the average value for the water absorption of each of three tested recycled aggregates and their standard deviation, with no homogeneous groups in this case and the recycled ceramic aggregate being the one with the highest absorption coefficient. This high-water absorption presented by recycled aggregates is one of their major drawbacks when it comes to being used as a raw material in the preparation of mortars and concretes since it directly affects the amount of mixing water and the subsequent appearance of humidity by capillarity, which in turn leads to a decrease in its mechanical properties [57,58]. Figure 8 shows a moderate dispersion of the results concerning the mean value, an idea that is contrasted with the low coefficient of variation reflected in Table 7. In the calculated confidence intervals, it can be observed that none of three types of recycled aggregates exceeds the value of 10%, set as the limit for the viability of this type of materials [34].

#### 3.1.7. Granulometric Analysis

**Figure 9:**
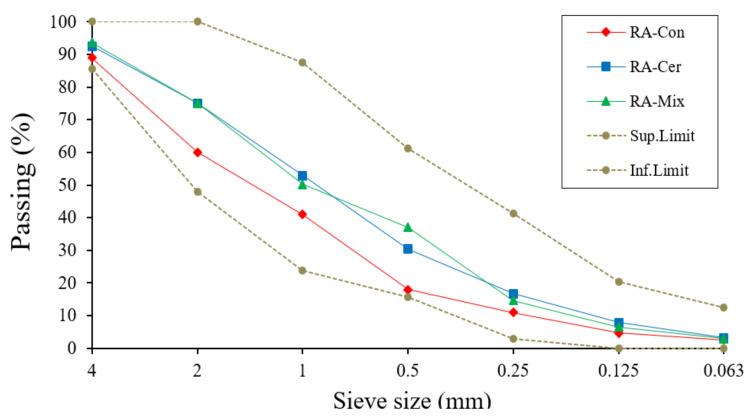
Fine recycled aggregates size distribution curve.

To determine the granulometry of recycled aggregates, the recommendations of the UNE-EN 933-1 standard [42] have been followed. This analysis consists of separating the particles by fractions of the same size and finding the percentage by weight of aggregate that enters each of the sizes. This is achieved by using a mechanical reciprocating sieve series of standard sieves with mesh sizes of 4, 2, 1, 0.5, 0.250, 0.125, 0.063 mm [24]. Ideally, for each aggregate, there are fractions of all sizes between the largest and smallest sieves. When this is fulfilled, it means that the aggregate has a continuous granulometry. There will be fewer segregation problems and it will be easier to apply it on-site [59]. In Figure 9, an example of the granulometric curves obtained for three types of recycled aggregates used in this study is shown.

As can be observed in Figure 9, the granulometry is continuous for three types of analyzed recycled aggregates and is established within the limits indicated by the reference regulations. Because of this continuous granulometry, the suitability of these residues can be considered as a raw material for the production of binding materials. This uniformity in the different sizes improves properties such as workability, compactness, and mechanical resistance of the mortars and concretes fabricated with them [60]. According to some authors, the use of these granulometric materials can improve the physical-mechanical properties of the concretes, while it is possible to mitigate the environmental impact linked to the better use of raw materials [61].

In summary, and as some authors affirm, recycled aggregates recovered from CDW can be considered more sustainable than currently used natural resources [62]. This is because, as has been proven, they have good physical properties and even better conditions as far as Life Cycle Assessment is concerned. In any case, these types of experimental studies contribute to improve the management of CDW and to understand better the benefits and characteristics of this type of recycled materials, trying to promote their use in the construction sector and reduce the consumption of raw materials from natural resources.

### 3.2. Chemical Characterization of Recycled Aggregates

#### 3.2.1. X-ray Diffraction

For the identification of the crystalline phases existing in the samples corresponding to three types of analyzed recycled aggregates, the X-ray diffraction technique was used. The results shown in Table 8 correspond to the mean value of the crystalline phases found in the analysis of three random samples of each type of tested aggregate. This analysis has to be understood as a guidance, as the composition of CDW can vary depending on its origin or the nature of raw materials it comes from. To conduct this test, a Siemens D5000 diffractometer with a graphite monochromator was used. The diffraction patterns have been measured using Cu-Kα (λ = 1.540598 Å). The results obtained for this test are shown in Table 8 and were analyzed using the EVA program of the Bruker company.

As can be seen in Table 8, which synthesizes the results obtained in the diffractograms made in each of the analyzed aggregate samples, the predominant crystalline phases for three types of studied aggregates have been quartz and calcite, as they have higher relative abundance. Moreover, in general, in the different samples, secondary crystalline phases were found that present an amorphous behavior, highlighting albite, sanidine, phlogopite, gypsum, and basanite. These results are following other studies conducted on this type of construction and demolition waste, where the higher gypsum content of recycled ceramic and mixed aggregates obtained as impurities after crushing and grinding of these CDWs stood out, mainly due to the good adhesion between the plaster and the ceramic surfaces where it is applied [63]. In addition, these compounds can negatively influence the durability of concrete structures that use these aggregates as raw materials.

#### 3.2.2. Thermogravimetric Analysis (TGA)

**Figure 10:**
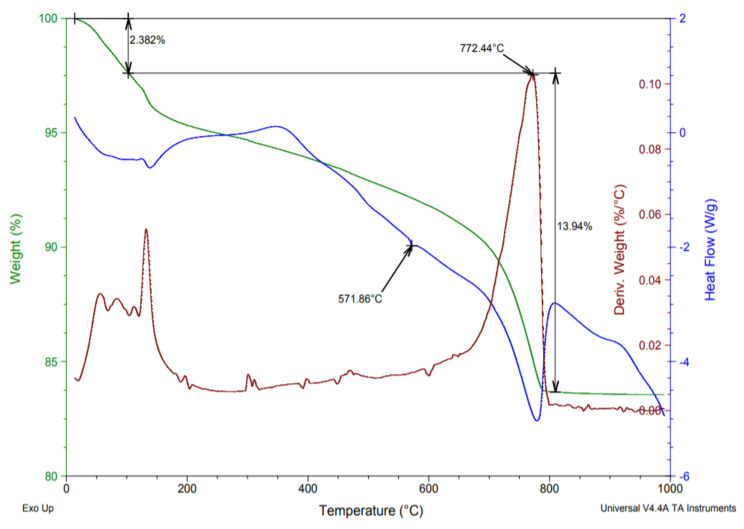
Example of a thermogram for recycled concrete aggregate sample.

To quantitatively analyze the composition and thermal stability of the aggregates, the thermogravimetry test has been conducted. This test is based on the measurement of the variation in mass changes as a result of a variation in the temperature at which the sample is subjected [64]. The results are expressed graphically by thermograms such as the one shown in Figure 10 which corresponds to the recycled concrete aggregate. The used equipment has been an SDT Q600 analyzer that allows a TGA Thermogravimetric Analysis and a DSC heat differential measurement to be performed simultaneously. Furthermore, to conduct this thermogravimetric test (ATG-DTA), the samples of the different aggregates were kept for five minutes at 100 °C to eliminate the initial humidity.

As can be seen from the thermogram in Figure 10, the mass loss and the derivative of the mass loss are present in the same temperature region. The first mass loss up to 110 °C corresponds to the initial moisture loss. Between 160 °C and 185 °C, the decomposition of the gypsum in the samples can be observed [65]. Around 570 °C a phase change is observed from αSiO_2_ (alpha-quartz) to βSiO_2_ (beta -quartz) and finally between 650 °C and 750 °C calcite decarbonization occurs.

On the other hand, Table 9 shows the percentages by weight for the calcite and the existing gypsum in the recycled aggregate samples according to the performed thermograms. It can be observed that the content in these raw materials is higher in aggregates from ceramic and mixed waste compared to concrete waste. These higher percentages in ceramic and mixed aggregates are caused by the greater amount of gypsum and calcite compounds that remain adhered to these aggregates after demolition and subsequent crushing [66].

#### 3.2.3. X-ray Fluorescence

X-ray spectrometry is a non-destructive qualitative and quantitative elemental analysis method based on the measurement of wavelengths or X-ray energy, emitted by the sample after being subjected to a high-energy primary radiation [67]. The spectrometer used has a radio anode, six analyzer crystals, flow and scintillation detectors and is capable of quantifying from traces (ppm) to 100% of the element in raw materials, ceramic materials, and glasses. The results obtained on average for each type of tested aggregate are shown in Table 10. As in the case of TGA, this test is a guidance which show average results obtained from the analysis of three random samples for each type of aggregate.

In each one of the tested samples, it has been observed that the recycled aggregates are mainly composed of silicates, with calcium and aluminum standing out. This characteristic becomes even more noticeable in the recycled ceramic aggregate if the percentage results obtained for the other two typologies are taken into account, and this is mainly due to the higher amount of clays present in these aggregates. Finally, it should be noted that the values of CaO and loss on calcination are within the ranges obtained by other investigations [68].

#### 3.2.4. Chloride and Total Sulfur Content

For the determination of the chlorides and total sulfur content of recycled aggregates, the recommendations of the UNE-EN 1744-1:2010+A1:2013 standard [69] have been followed. In the case of chloride content, the Volhard method has been used, consisting of adding an excess of a silver nitrate solution and another solution containing chloride ions, in such a way that the amount of silver nitrate solution that has not reacted, is titrated back with a standard thiocyanate solution, using an ammonium iron III sulfate solution as an indicator [70]. To determine the total sulfur content, the recycled aggregate samples are treated with hydrogen peroxide and hydrochloric acid to transform all the sulfur compounds into sulfates. Subsequently, all sulfates precipitate in the form of barium sulfate and are weighed [71]. Table 11 shows, orientatively, the mean of the six values obtained for each type of analyzed aggregate.

As can be observed in Table 11, the content of water-soluble chlorides presents percentages lower than 0.06% set as a maximum for construction aggregates to be used in masonry mortars in the UNE-EN 988-2 standard [72]. On the other hand, the total sulfur content observed in the tested samples is also less than the 1% limit established by the UNE-EN 13,139 [45] standard. Only in one of the tested samples was the value of 0.9 exceeded. The results correspond to those obtained in other previously conducted investigations [73].

## 4. Discussion

In this research work, the main physicochemical properties of three different types of construction and demolition waste have been analyzed: recycled ceramic aggregate, recycled concrete aggregate, and mixed recycled aggregate, monitoring the recycled aggregate manufacturing process conducted by two plants of Construction and Demolition Waste management located in the Community of Madrid for a period of two years. The paper presents a high interest for professionals of the construction sector, who can see their attempts to use this typology of recycled materials reinforced. It contributes to achieving sustainable waste management and efficient use of natural resources, following the Goals for Sustainable Development in the 2030 Agenda.

The most relevant conclusions obtained from the physical characterization of the aggregates are listed below:

- It has been possible to corroborate that a high content of fines is produced during the process of elaboration of recycled aggregates, and, as these aggregates reach a high modulus of fineness. Regarding the performed statistical analysis, it can be observed that there are no significant differences in terms of the content in fines for three types of recycled aggregates. The fineness modulus of the RA–Mix presents significantly higher values for this property compared to the rest of tested aggregates at 99.7% confidence.
- The coefficient of friability of the aggregates is also impaired because of the recycling process. Once the statistical analysis has been conducted, it has been observed that there are significant differences for three types of analyzed aggregates, being the recycled concrete aggregate the one that shows the best properties.
- Regarding the relative density and overall density values obtained, it has been possible to verify that RA–Con has obtained significantly higher values compared to the other two recycled aggregates. These higher density values make it the optimal aggregate for the preparation of masonry mortars and concrete if higher mechanical resistance is to be achieved.
- Regarding the water absorption, all the aggregates have presented statistically significant differences, being the RA–Cer the recycled aggregate that has presented a higher percentage for this property, and the RA–Con is one which shows the lowest values. However, despite their high absorption, these recycled aggregates can be used as raw material for the manufacture of mortars and concrete.
- Lastly, it has been verified that three types of recycled aggregates used in this research present a continuous granulometry within the limits established by the reference regulations for the preparation of masonry mortars. This continuous granulometry favors the workability of the binder material mixture and has a positive effect on its mechanical properties.

On the other hand, the conclusions obtained from the chemical characterization of the aggregates are listed as follows:

- From the analysis of X-ray diffraction for three types of recycled aggregates, it has been observed that the predominant crystalline phases are quartz and calcite, as they have higher relative abundance. Although other secondary crystalline phases such as albite, sanidine, phlogopite, gypsum, and baseite have been found.
- Regarding the thermogravimetric analysis, it has been observed that the content of calcite and gypsum compounds is higher in the aggregate samples from ceramic waste. These results show that RA–Cer is the least suitable recycled aggregate for the fabrication of structural concretes.
- With the quantitative and qualitative analysis of the composition of the aggregates conducted by employing X-ray fluorescence, it has been possible to verify that these recycled aggregates are mainly composed of calcium and aluminum silicates.
- Finally, the content of water-soluble chlorides and total sulfur presented percentages are lower than 0.06% and 1%, respectively, values set as maximum admissible in construction aggregates.

## 5. Conclusions

As a general conclusion of the research, it can be stated that three types of analyzed recycled aggregates have presented physicochemical characteristics within the limits set by the reference regulations for aggregate fractions between 0.063 and 4 mm in all the calculated confidence intervals. Moreover, it has been observed how these aggregates present sufficient homogeneity in their properties over time, which would further promote the use of these raw materials in the construction sector and contribute to the achievement of efficient management construction and demolition waste. In any case, the use of this type of recycled aggregates significantly reduces the environmental impact caused by the construction sector [74], although it would be interesting to consider effects such as the emissions generated by the transport of these raw materials to work. Additionally, recycled concrete aggregate has been positioned as the most suitable recycled raw material for the manufacture of mortars. This affirmation is supported by its good chemical properties, its higher density which would result in a higher compressive strength of the hardened mortars and its lower water absorption coefficient which results in a lower demand for mixing water.

Finally, as limitations of this work and future lines of research, it is proposed to conduct complementary tests to those used in this research, such as tests of water-soluble and acid-soluble sulfate content, water-soluble salts, percentage of organic material, plasticity, or assigned classification type. All of them would allow a more exhaustive and complete characterization of the recycled aggregates that are currently used in the Community of Madrid and would serve as a reference for professionals in the sector who are interested in the use of this type of raw materials. In addition, it would be advisable to extend the sample used to conduct the chemical characterization tests, since these properties are particular to each type of aggregate depending on its origin. For this reason, the results obtained in this section should be interpreted as a real approximation to the problem, which should be customized for each specific investigation.

## Figures and Tables

**Table 1:** Physical properties characterization of recycled aggregates.

Type	RA–Con		RA–Cer		RA–Mix	
Test	W Statistic	*p*-Value	W Statistic	*p*-Value	W Statistic	*p*-Value
Fine content	0.928636	0.092688	0.968940	0.641494	0.974845	0.779908
Fineness modulus	0.938668	0.156609	0.959768	0.440422	0.974616	0.774718
Friability	0.963739	0.522800	0.958691	0.419649	0.942720	0.192893
Set density	0.949215	0.267455	0.920243	0.059527	0.963892	0.526158
Relative density	0.979905	0.884980	0.933410	0.119093	0.958022	0.407095
Absorption	0.976486	0.706861	0.936322	0.050249	0.950606	0.144408

**Table 2:** Statistical Analysis of fines content.

ANOVA				Typical Deviation (%)	Variation Coefficient	Confidence Interval (99.7%)
RA	Quantity	Average (%)	Homogeneous Groups			
Con	24	5.17	X	0.964	0.186	[2.28; 8.10]
Mix	24	5.48	X	0.799	0.146	[3.08; 7.88]
Cer	24	5.63	X	0.796	0.141	[3.24; 8.02]

**Table 3:** Statistical Analysis of Fineness Module.

ANOVA				Typical Deviation (%)	Variation Coefficient	Confidence Interval (99.7%)
RA	Quantity	Average (%)	Homogeneous Groups			
Con	24	4.84	X	0.614	0.127	[3.00; 6.68]
Mix	24	5.33	XX	0.565	0.106	[3.64; 7,03]
Cer	24	5.02	X	0.469	0.093	[3.61; 6.43]

**Table 4:** Statistical Analysis of Friability Coefficient.

ANOVA				Typical Deviation (%)	Variation Coefficient	Confidence Interval (99.7%)
RA	Quantity	Average (%)	Homogeneous Groups			
Con	24	24.60	X	0.682	0.028	[22.55; 26.65]
Mix	24	27.10	X	0.797	0.029	[24.71; 29.49]
Cer	24	26.26	X	0.660	0.025	[24.28; 28.24]

**Table 5:** Statistical Analysis of Set Density.

ANOVA				Typical Deviation (%)	Variation Coefficient	Confidence Interval (99.7%)
RA	Quantity	Average (%)	Homogeneous Groups			
Con	24	1300.8	X	35.3	0.027	[1194.9; 1406.6]
Mix	24	1238.2	X	43.1	0.035	[1108.9; 1367.5]
Cer	24	1258.1	X	36.5	0.029	[1148.7; 1367.6]

**Table 6:** Statistical Analysis regarding Relative Density.

ANOVA				Typical Deviation (%)	Variation Coefficient	Confidence Interval (99.7%)
RA	Quantity	Average (%)	Homogeneous Groups			
Con	24	2210.3	X	38.3	0.017	[2095.6; 2325.1]
Mix	24	2141.8	X	26.0	0.012	[2063.8; 2219.7]
Cer	24	2136.1	X	25.5	0.012	[2059.6; 2212.6]

**Table 7:** Statistical Analysis regarding Water Absorption.

ANOVA				Typical Deviation (%)	Variation Coefficient	Confidence Interval (99.7%)
RA	Quantity	Average (%)	Homogeneous Groups			
Con	36	6.35	X	0.57	0.089	[4.66; 8.05]
Mix	36	6.70	X	0.68	0.102	[4.65; 8.75]
Cer	36	7.30	X	0.58	0.079	[5.56; 9.04]

**Table 8:** Analysis by X-ray diffraction. The relative abundance (*; **; ****; *****) of each type of mineral crystalline phase was found.

Mineral Phase	Calcite	Quartz	Gypsum	Sanidine	Phlogopite	Basanite
RA–Con	****	*****	*	*	*	*
RA–Cer	****	*****	**	**	**	*
RA–Mix	****	*****	**	**	**	*

**Table 9:** Quantification of gypsum and calcite in recycled aggregates by TGA.

Mineral	RA–Con	RA–Mix	RA–Cer
% Calcite	17.12	17.53	18.90
% Gypsum	4.14	4.35	5.38

**Table 10:** X-Ray Fluorescence test.

Samples	Al_2_O_3_	CaO	Fe_2_O_3_	K_2_O	MgO	SiO_2_	MnO	TiO_2_	SO_3_	P_2_O_5_	NaO_2_	I. Loss (%)
RA–Con	6.22	11.43	1.52	2.15	0.65	66.9	0.035	0.14	-	0.10	0.38	9.35
RA–Mix	6.88	10.38	1.27	2.20	0.57	68.4	0.025	0.13	-	0.11	0.24	9.50
RA–Cer	10.26	16.85	2.84	2.38	1.75	43.7	-	0.35	4.32	0.12	0.80	16.25

**Table 11:** Chemical characteristics of recycled aggregates.

Test	Regulation	RA–Con	RA–Mix	RA–Cer
Chloride content (%)	UNE–EN 1744-1	0.0054	0.0026	0.0041
Total sulfur (%)	UNE–EN 1744-1	0.69	0.81	0.92

## Data Availability

Not applicable.
